# Aberrant glycosylation in colorectal cancer with genomic and epigenomic alterations

**DOI:** 10.18632/oncotarget.26292

**Published:** 2018-11-02

**Authors:** Hirokazu Okayama, Masaru Noda, Koji Kono

**Affiliations:** Department of Gastrointestinal Tract Surgery, Fukushima Medical University School of Medicine, Fukushima, Fukushima, Japan

**Keywords:** colorectal cancer, glycosylation, Tn-antigen, DNA mismatch repair deficiency (dMMR), CpG island methylator phenotype (CIMP)

The molecular pathogenesis of colorectal cancer (CRC) is highly heterogeneous that involves the accumulation of genetic and epigenetic changes, resulting in diverse clinical outcomes. It has been recognized that genetic abnormalities in CRCs can be commonly grouped into two distinct categories conferring particular phenotypes, chromosomal instability (CIN) and hypermutated [1, 2]. The majority of CRCs (~85%) follows the CIN pathway exhibiting a high frequency of somatic copy number alterations and marked aneuploidy, but microsatellite stable (non-hypermutated). CIN tumors are often accompanied by *KRAS* oncogene mutations and inactivation of *TP53* tumor suppressor gene [1, 2]. Hypermutated tumors, accounting for approximately 15% of CRCs, develop through the fundamentally different pathway that manifest the high-level microsatellite instability (MSI-H) phenotype, caused by deficiency in the DNA mismatch repair (MMR) system [1, 2]. In sporadic CRCs, deficient MMR (dMMR) is commonly due to promotor hypermethylation of the MMR gene, *MLH1*, often associated with CpG island methylator phenotype (CIMP). CIMP-high tumors (~20%) overlap greatly with MSI-H CRCs and some non-hypermutated CRCs, and those phenotypes frequently carry oncogenic *BRAF* mutations and correlate with proximal tumor location, female gender and poor differentiated and mucinous histology. The prognostic impact of MSI-H depends on disease stage, but both CIMP and mutant *BRAF* are known to be associated with poor survival [3].

Glycosylation is the most frequent post-translational modification reaction in mammalian cells that play a pivotal role in the regulation of diverse physiological processes. Glycosylation is catalyzed by enzymatic reaction of a large number of glycosyltransferases, the mechanism of which involves sequential addition of single sugar residues to target molecules along with chemical modifications and branching, resulting in glycan elongation with a vast array of decorated structures [4]. During malignant transformation and tumor progression, cell surface glycan profiles change dramatically. Altered glycan structures that preferentially appear on cancer cell surface are known to be responsible for distinct biological functions and unique tumor phenotypes, which can be attributed to dysregulation of multiple glycosyltransferases at the transcriptional level. Two principal mechanisms, including “incomplete synthesis” and “neo-synthesis”, have been postulated to control glycan alterations in cancer cells [4, 5]. The expression of certain glycosyltransferases genes can be induced at advanced stages, resulting in the de novo expression of cancer-antigens, called neo-synthesis process. In early stages of tumorigenesis, transcriptional repression of some genes, encoding glycosyltransferases, through epigenetic silencing can lead to the synthesis of truncated or shortened glycan structures, namely, incomplete synthesis [6].

In our recent study, we attempted to identify molecular subclasses associated with glycosyltransferase gene expression profiles [7]. We assembled an extensive number of CRC samples and cohorts, by integrating available information on clinicopathological and genomic features, including mutations, MMR status, methylation profiles, as well as the use of cancer precursor tissues. We discovered a transcriptional subtype characterized by a set of glycosyltransferase genes, displaying poor prognosis, poorly differentiated and mucinous histology, proximal tumor location, and dMMR. Notably, this subtype consistently demonstrated decreased expression levels of *GALNT6* gene, which encodes one of the polypeptide GalNAc transferase (ppGalNAc-T) family enzymes involved in the initial step of O-glycan synthesis. Downregulation of *GALNT6* seemed to occur during adenoma-to-carcinoma transition, possibly through epigenetic mechanisms, where *GALNT6* was highly upregulated in adenomas but was subsequently methylated and decreased in some carcinomas that frequently exhibited dMMR. Immunohistochemistry analysis also demonstrated that almost all adenomas were positive for GALNT6 staining, whereas approximately 15% of carcinomas showed loss of GALNT6 protein expression. The lack of GALNT6 protein was observed in more than 50% of dMMR tumors, but it was less frequent (<12%) in proficient MMR CRCs. *GALNT6* depletion *in vitro* induced the cell surface expression of a truncated O-glycan, Tn-antigen, identified by lectin microarray followed by flow cytometric analysis. Correspondingly, treatment with demethylating agent reactivated the expression of *GALNT6* along with diminished cell surface Tn-antigen in CRC cells. Those findings in our study were highly consistent with the concept of incomplete synthesis that glycan elongation in nonmalignant cells are impaired upon malignant transformation by epigenetic silencing of glycosyltransferases, resulting in cancer-associated glycan truncation [4]. Indeed, Tn-antigen was previously reported as a cancer-antigen, correlating with poorly-differentiated or mucinous adenocarcinoma and poor survival in CRC [4, 5, 8]. Our study also revealed that decreased levels of *GALNT6* mRNA and loss of GALNT6 protein were each associated with poor survival, and its prognostic value was particularly found in patients with stage III CRC [7]. Intriguingly, a recent study stated that elevated expression of Tn-antigen (MGL ligands) recognized by lectin MGL-binding was positively correlated with mutant *BRAF*, and had significant impact on poor prognosis in patients with stage III CRC [9]. Our study also showed that not only MSI-H but CIMP and *BRAF* mutations were enriched in tumors with decreased *GALNT6* expression, despite the insufficient data availability in many of the cohorts we used. Collectively, tumors exhibiting decreased *GALNT6*, CIMP-high, mutant *BRAF* and increased Tn-antigen seem to share similar clinicopathological characteristics, including proximal location, poorly-differentiated or mucinous histology and poor survival outcomes, which overlap imperfectly with dMMR/MSI-H phenotype. Likewise, the prognostic impact of GALNT6 expression might be at least in part attributed to CIMP and/or *BRAF* status. Although the underlying driver biology remains inconclusive, CIMP might be one of the key molecular pathways that can explain the epigenetic silencing of *GALNT6*. In conclusion, our study supports the role of incomplete glycan synthesis in colorectal carcinogenesis. Future studies would be required to address the complex mechanisms of regulating Tn-antigen expression, *GALNT6* and other glycosyltransferases, particularly in relation to genomic and epigenomic characteristics of CRC.
